# Tumor suppressor gene RBM5 delivered by attenuated *Salmonella* inhibits lung adenocarcinoma through diverse apoptotic signaling pathways

**DOI:** 10.1186/1477-7819-11-123

**Published:** 2013-05-31

**Authors:** Chen Shao, Baoxue Yang, Lijing Zhao, Song Wang, Jie Zhang, Ke Wang

**Affiliations:** 1 Department of Respiratory Medicine, the Second Affiliated Hospital of Jilin University, 18 Ziqiang Street, Changchun, Jilin 130041, China; 2 Department of Digestive Medicine, China-Japan Union Hospital of Jilin University, 126 Xiantai Street, Changchun, Jilin 130033, China; 3 Department of Pathophysiology, Norman Bethune College of Medicine of Jilin University, 18, Zhiqiang Street, Changchun, Jilin 130021, China; 4 Department of Pharmacology, School of Basic Medical Sciences of Peking University, 38 Xueyuan Road, Beijing 100191, China; 5 Department of Urinary Surgery, the First Affiliated Hospital of Jilin University, 71 Xinmin Street, Changchun, Jilin 130041, China

**Keywords:** RBM5, Lung adenocarcinoma, Apoptosis, A549, Xenograft mice model, Attenuated *Salmonella*

## Abstract

**Background:**

RBM5 (RNA-binding motif protein 5, also named H37/LUCA-15) gene from chromosome 3p21.3 has been demonstrated to be a tumor suppressor. Current researches *in vitro* confirm that RBM5 can suppress the growth of lung adenocarcinoma cells by inducing apoptosis. There is still no effective model *in vivo*, however, that thoroughly investigates the effect and molecular mechanism of RBM5 on lung adenocarcinoma.

**Method:**

We established the transplanted tumor model on BALB/c nude mice using the A549 cell line. The mice were treated with the recombinant plasmids carried by attenuated *Salmonella* to induce the overexpression of RBM5 in tumor tissues. RBM5 overexpression was confirmed by immunohistochemistry staining. H&E staining was performed to observe the histological performance on plasmids-treated A549 xenografts. Apoptosis was assessed by TUNEL staining with a TUNEL detection kit. Apoptosis-regulated genes were detected by Western blot.

**Results:**

We successful established the lung adenocarcinoma animal model *in vivo*. The growth of tumor xenografts was significantly retarded on the mice treated with pcDNA3.1-RBM5 carried by attenuated *Salmonella* compared to that on mice treated with pcDNA3.1. Overexpression of RBM5 enhanced the apoptosis in tumor xenografts. Furthermore, the expression of Bcl-2 protein was decreased significantly, while the expression of BAX, TNF-α, cleaved caspase-3, cleaved caspase-8, cleaved caspase-9 and cleaved PARP proteins was significantly increased in the pcDNA3.1-RBM5-treated mice as compared to that in the control mice.

**Conclusions:**

In this study, we established a novel animal model to determine RBM5 function *in vivo,* and concluded that RBM5 inhibited tumor growth in mice by inducing apoptosis. The study suggests that although RBM5’s involvement in the death receptor-mediated apoptotic pathway is still to be investigated, RBM5-mediated growth suppression, at least in part, employs regulation of the mitochondrial apoptotic pathways.

## Background

Lung cancer is the leading cause of cancer death worldwide, with over a million deaths annually [1,2]. The five-year survival rate has not improved dramatically throughout the past three decades despite innovations in diagnostic testing, surgical technique, and development of new chemotherapeutic agents [3,4]. Lung cancer histology is heterogeneous. Small cell lung carcinomas (SCLCs) account for 20% of all lung cancers, and the remaining 80% consists of non-small cell lung carcinomas (NSCLCs) which, in turn, is further subclassified into 40% adenocarcinoma (AC), 40% squamous cell carcinoma (SCC) and 20% large cell carcinoma. Delineating genetic alterations specific to each subtype of lung cancer may be the most effective way of discovering molecular markers for early detection and developing better individualized treatment.

The loss of tumor suppressor gene (TSG) function is a critical step in the pathogenesis of human lung cancer. The earliest premalignant chromosomal aberration in human lung cancers is allele loss within the short arm of chromosome 3 at 3p21.3 [5]. This loss of heterozygosity occurs in practically all (>95%) SCLC tumors, the majority (>70%) of NSCLC tumors [6-8]. Deletion at chromosome 3p21.3 is also the most frequent genetic alteration identified in lung cancer. The region contains 19 TSGs [9], most of which demonstrate varying degrees of tumor suppressor activity (related to the control of processes such as cell differentiation, proliferation, signal transduction, and apoptosis). It has been suggested that all function together as a large, integrated, biologically functionally diverse tumor suppressor unit [7].

RNA-binding motif protein 5 (RBM5, previously referred to as g15, LUCA-15, and H37) that maps to one end of this 19-gene deletion breakpoint, is an RNA-binding protein that has the ability to modulate apoptosis and cell cycle arrest through pre-mRNA splicing of multiple target genes, such as p53 [10-17]. Overexpression of RBM5, which is also involved in the regulation of alternative splicing, showed the function of inhibiting tumor growth and reducing the metastatic potential [18-20]. In addition, multiple protein isoforms of RBM5 exist, each possessing apoptosis modulatory activity, a function consistent with tumor suppressor activity. For the inhibitory effect of RBM5 on lung cancer, in current studies, it is reported that RBM5 modulates apoptosis by regulating the alternative splicing of apoptosis-associated pre-mRNAs, such as CASP2 and FAS/CD95 [2,21]. In Oh’s study, they found RBM5 could significantly inhibit the growth of A549 non-small cell lung cancer cells by inducing apoptosis, which is associated with upregulation of the proapoptotic protein BAX, increased release of mitochondrial cytochrome C into the cytosol and increased activation of caspases 9 and 3 [12,14,16]. Most of the researches, however, were restricted to the cell line *in vitro*. In addition, in most of these approaches, the therapeutic agents could not reach the tumors in effective doses, or were distributed to unwanted sites and degraded by nucleases resulting in limited antitumor effects. Currently, there is not an effective animal model for further *in vivo* exploration of the function and mechanism of RBM5 on primary lung cancer. Furthermore, how to deliver a vector overexpressing RBM5 to the tumor-specific part is the key point for the study *in vivo*. Fortunately, the discovery that genes in bacterial vectors can be functionally transferred to mammalian cells suggests that it might be possible to use bacterial vectors as vehicles for gene therapy. Genetically modified nonpathogenic bacteria have been used as potential antitumor agents, to either elicit direct tumoricidal effects or deliver tumoricidal molecules [22]. Bioengineered attenuated strains of *Salmonella enterica* serovar Typhimurium have been shown to preferentially accumulate by >1000-fold in tumors compared with normal tissues and to become homogeneously dispersed in the tumor tissues [23]. These attenuated bacteria have been proven safe in mice, pigs and monkeys when administered intravenously [22].

In this study, we first establish an effective animal model using BALB/c nude mice treated with attenuated *Salmonella* as a vector carrying plasmids to explore the molecular mechanisms involved in the antitumor effect induced by RBM5 on lung adenocarcinoma.

## Methods

### Cell line and plasmids

The A549 cell line was purchased from the American Tissue Type Collection (Manassas, VA, USA). Cells were grown in RPMI 1640 supplemented with 10% fetal bovine serum as previously described [24]. Plasmids of pcDNA3.1 and pcDNA3.1-RBM5 were generously provided by Dr. Leslie Sutherland of the Research Program, Northeast Cancer Centre, Health Sciences North, Ontario, Canada.

### Electrotransfection of RBM5 into competent attenuated *Salmonella* cells

Competent *S. enteric*a ser. Typhimurium cells (competence) were obtained from the China-Japan Union Hospital of Jilin University in China. The competence were mixed with 1 μg pcDNA3-RBM5 or 1 μg pcDNA3 plasmids and cooled for 15 minutes on ice, Then the DNA could be transfected into the competence under the conditions as follows: C = 25 μF, PC = 200 ohm, V = 1.25 kV (12.5 kV/cm). Then the competence with DNA should be quickly transferred into LB Ager medium for proliferation at 37°C. The recombinant attenuated *Salmonella* strains carrying plasmids were stored at −80°C for later use.

### Establishment of A549 xenografts

BALB/c athymic nude male mice (nu/nu; 6 weeks) were purchased from the Institute of Zoology, Chinese Academy of Sciences, Beijing. Use of animals was in accordance with Animal Care guidelines and the protocol was approved by Jilin University Animal Care Committee. A549 cells were washed and resuspended in phosphate-buffered saline (PBS). The suspension (5 × 10^6^ cells in 150 μL per mouse) was inoculated subcutaneously into the right flanks of nude mice. The sizes of the tumors were measured using calipers starting from day 7 after cell injection until day 42.

### Gene treatment

The tumor-bearing mice were divided randomly into two groups (six mice per group) at day 21 after cell injection. The mice were treated at day 28 and 35 respectively through a tail mainline as follows: (a) control group (attenuated *Salmonella* carrying pcDNA3.1); (b) RBM5 group (attenuated *Salmonella* carrying pcDNA3.1-RBM5) (10^8^ colony-forming units (CFU) per 50 μL PBS). Then the tumors were measured using calipers every 3.5 days until day 42. The data were plotted using the Kaplan-Meier method to analyze the tumor growth curves. The wet weight and size of the tumors were measured when the mice were sacrificed on day 42. The tumor blocks were taken out, a part of which was fixed in formalin for hematoxylin and eosin (H&E) staining and immunohistochemistry analysis, and another part of which was snap frozen in liquid nitrogen immediately for Western blot and terminal deoxyuridyl transferase (TdT)-mediated deoxyuridine 5'-triphosphate (dUTP) nick-end labeling (TUNEL) assay (Roche, Penzberg, Germany).

### TUNEL staining of apoptotic cells

The tissue sections of xenografts from the nude mice were dewaxed and hydrated and then were subjected to TUNEL staining following the instructions provided by the manufacturer (Promega, Madison, WI, USA). Four fields were chosen randomly and analyzed. The apoptotic index was defined as follows: apoptotic index (%) = 100× apoptotic cells/total tumor cells.

### H&E and immunohistochemistry staining

Tumors treated with recombinant *Salmonella* strains carrying different plasmids were H&E stained. Immunohistochemistry analyses were carried out as described previously [25,26]. Anti-human rabbit RBM5 antibody was purchased from Abcam (Cambridge, MA, USA).

### Protein extraction and Western blot

Total proteins from both tumor tissues were extracted according to the previous study [27]. Protein samples (30 ug) were then separated by SDS-PAGE and transferred onto a PVDF membrane (Millipore, Bedford, MA, USA). The primary antibodies were rabbit anti-human RBM5, Bcl-2, Bax, TNF-α, cleaved PARP, cleaved caspase-3, cleaved caspase-8, cleaved caspase-9 and β-actin antibodies from Abcam (Cambridge, MA, USA). The second antibody was a goat anti-rabbit IgG-HRP from Santa Cruz Biotechnology (Santa Cruz, CA, USA). Western blot was carried out as previously described [27]. The protein bands were visualized by SuperSignal West Pico Chemiluminescent Substrate (Thermo Scientific Pierce, Rockford, IL, USA), and the membranes were subjected to X-ray autoradiography. Band intensities were determined with Quantity One software (Bio-Rad, Hercules, CA, USA). Furthermore, we confirmed the reproducibility of the experiments at least three times.

### Statistical analysis

Statistical comparisons were performed using analysis of variance to determine the significance of differences observed between different treatment groups. Values are expressed as mean ± standard deviation (SD) from at least three separate experiments in which each experiment had six samples per treatment group and differences were considered significant at a *P* value of <0.05.

## Results

### Overexpression of RBM5 inhibited tumor growth in A549 xenograft BALB/c nude mice

**Figure 1:**
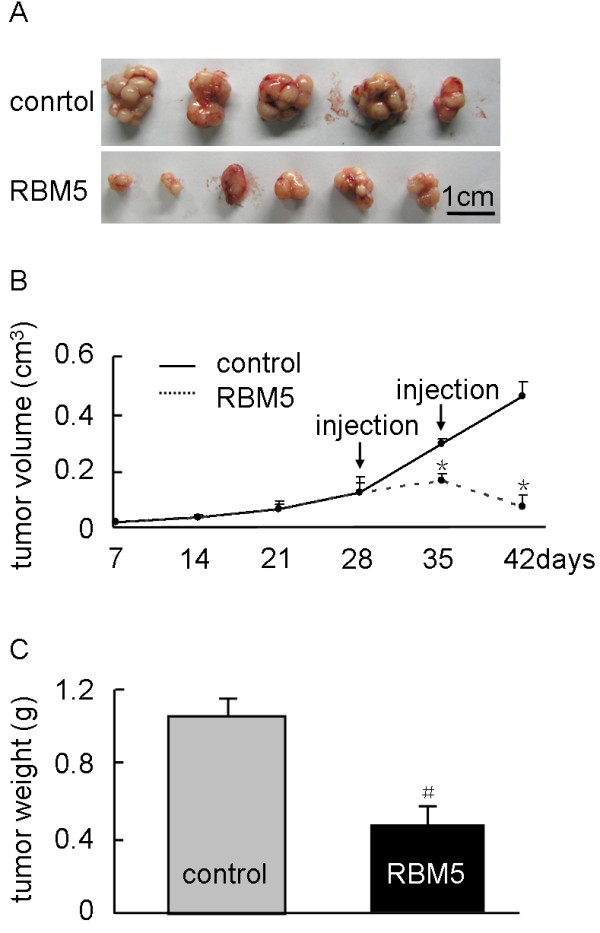
**RBM5 inhibits tumor growth *in vivo*.** Tumor-bearing mice were treated with attenuated *Salmonella* carrying pcDNA3.1 or pcDNA3.1-RBM5 by injection two times (on day 28 and 35). **(A)** Comparison of tumor sizes in two groups on day 42 after implantation. **(B)** Tumor growth curve. Tumor sizes were measured two times every week from day 7 to 42 after implantation. **(C)** Tumor wet weights were measured when the mice were sacrificed on day 4 after implantation. *indicates significant difference as compared to the control (*P* <0.05); **indicates significant difference as compared to the control (*P* <0.01). RBM5, RNA-binding motif protein 5.

The potential therapeutic effect on lung adenocarcinoma xenograft growth by RBM5 was examined in tumor-bearing mice treated with bacteria carrying RBM5 plasmid (Figure 1A). Dynamic tumor growth was monitored from day 7 to day 42 after injection. We showed that, while the sizes of the tumor xenografts between RBM5 and control groups were similar before day 28, the growth of tumor xenografts in the mice treated with RBM5 retarded after day 28 (Figure 1B). In addition, the weight of the tumor xenografts from mice treated with RBM5 became significantly lighter than that in the control group at day 42 when the mice were sacrificed (Figure 1C). This result suggested that accumulative and stable expression of RBM5 in A549 xenograft BALB/c nude mice significantly retarded the tumor growth rate *in vivo*. Moreover, we established a novel animal model using BALB/c nude mice treated with attenuated *Salmonella* as a vector carrying plasmids to determine RBM5 function *in vivo*.

### Analysis of apoptosis induced by RBM5 in A549 xenograft BALB/c nude mice

**Figure 2:**
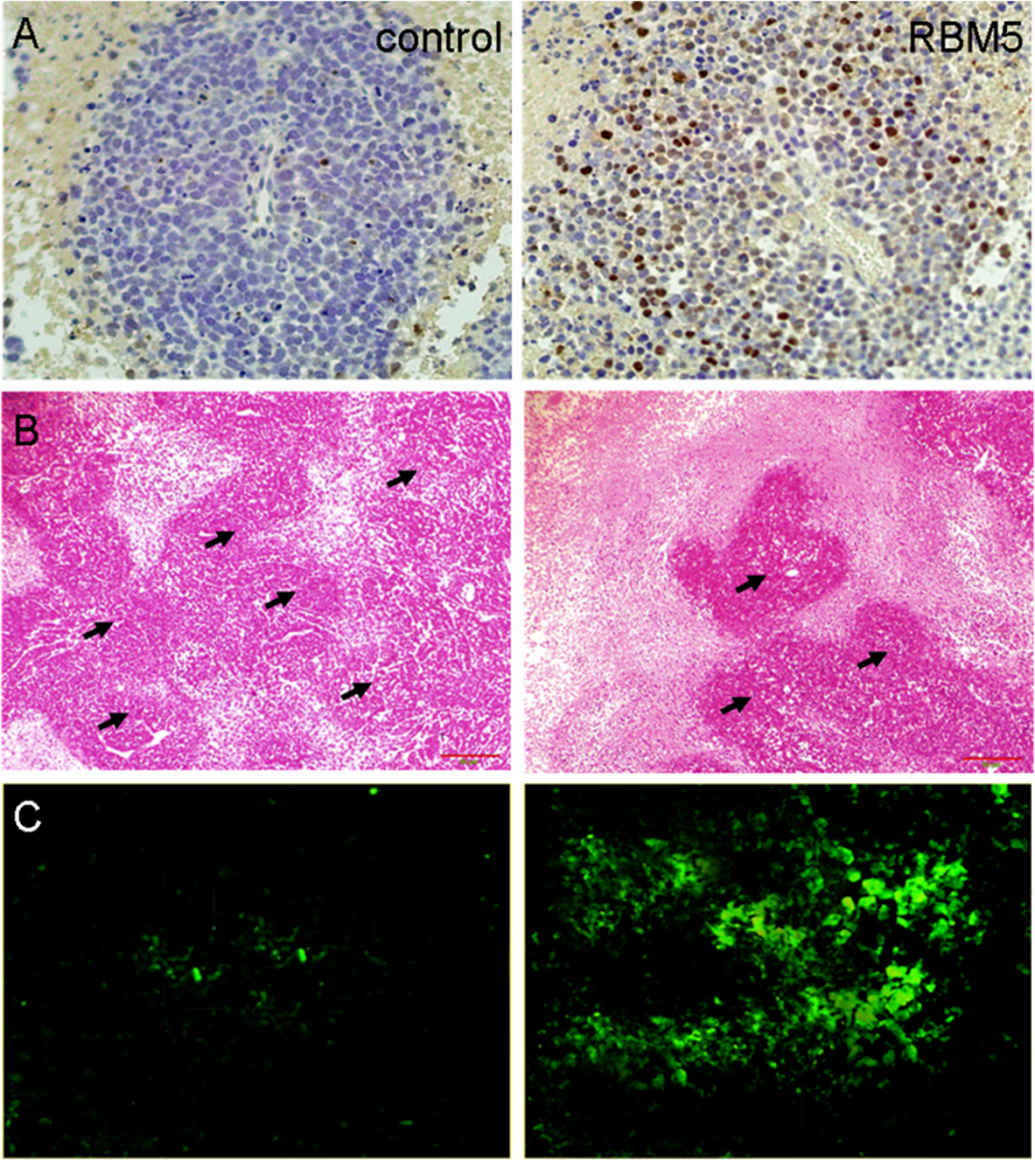
**H&E, immunohistochemistry and TUNEL assay. (A)** Immunohistochemistry staining for RBM5 (400×). **(B)** H&E staining (200×). ‘**↑**’ indicates cancer nest. **(C)** RBM5-induced apoptosis shown by TUNEL staining (400×). Tissue sections were treated with terminal deoxynucleotidyl transferase to incorporate fluorescein-deoxyuridine 5'-triphosphate (dUTP) to nicked DNA of apoptotic cells. Apoptotic cells exhibiting a strong nuclear green fluorescence were detected by fluorescence microscopy. Adjacent light microscopic photos showed a representative area from each group showing equal cell density and fixation. RBM5, RNA-binding motif protein 5.

To determine the potential mechanism of tumor growth inhibition *in vivo*, A549 xenograft treated with pcDNA3.1 control or pcDNA3.1-RBM5 were excised and analyzed by immunohistochemistry, H&E, and TUNEL staining. First, the overexpression of RBM5 was confirmed by immunohistochemistry staining. As shown in Figure 2A, RBM5 expression was significantly higher in the tumors of mice treated with pcDNA3.1-RBM5, compared to that in the tumors of mice treated with pcDNA3.1. This result suggested that the RBM5 protein was efficiently delivered and overexpressed by attenuated *Salmonella in vivo*.

We next employed H&E staining to observe the histopathological performance on A549 xenografts. The result showed that there were a large number of cancer nests in the control group (Figure 2B), and the tumor tissue could survive in a good state. It could be seen, however, in the RBM5 group, that the number of cancer nests significantly decreased, and the regions of necrosis were much larger than those in the control group (Figure 2B).

To explore the contribution of cell death and tumor retardation induced by RBM5, we performed TUNEL staining analysis to detect apoptotic cells in A549 xenografts. As shown in Figure 2C, approximately more than ten times apoptotic cells were observed in the RBM5 group compared with those in the control group, which indicated that RBM5 inhibited A549 xenograft tumor growth by inducing apoptosis.

### Analysis of mitochondrial and TNF-α related apoptotic pathways in RBM5 mediated-apoptosis

**Figure 3:**
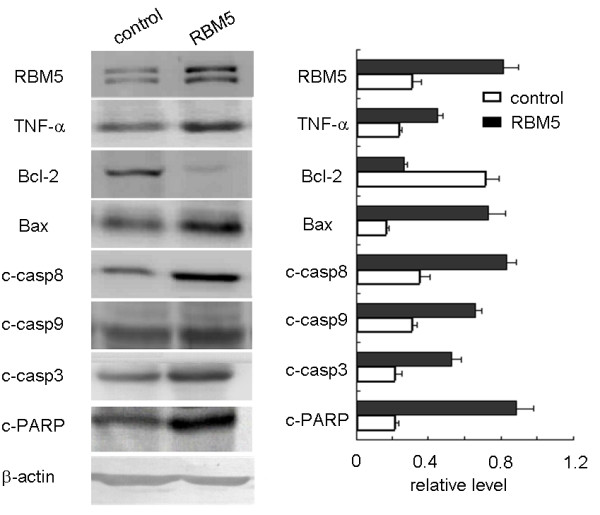
**Differential expression of apoptosis regulatory proteins associated with RBM5 overexpression.** Western blot analysis was performed to compare expression levels of various apoptosis-related proteins between the RBM5 and control groups. Among them, Bcl-2 expression was found to be decreased and RBM5, TNF-α, Bax, cleaved caspase-3, cleaved caspase-8, cleaved caspase-9 and cleaved PARP increased in the RBM5 group compared with the control. β-actin was used as a protein-loading control. RBM5, RNA-binding motif protein 5.

To further characterize the molecular mechanisms of the RBM5-mediated apoptotic pathways, we investigated the influence of mitochondrial apoptosis signaling and death receptors that characteristically initiate signaling. We examined the expression of some apoptosis-related genes including RBM5, Bcl-2, Bax, and TNF-α, cleaved caspase-3, cleaved caspase-8, cleaved caspase-9 and cleaved PARP. We observed that the expression of Bcl-2 protein was decreased significantly when RBM5 was overexpressed, while the expression of Bax, TNF-α, cleaved caspase-3, cleaved caspase-3, cleaved caspase-8, cleaved caspase-9 and cleaved PARP proteins were significantly increased in the RBM5 group as compared to that in the control cells (Figure 3), which suggested that, although involvement of RBM5 in the death receptor-mediated apoptotic pathway remains to be investigated in depth, RBM5- mediated growth suppression, at least in part, employs regulation of the mitochondrial apoptotic pathways.

## Discussion

In recent years, lung cancer has become one of the leading causes of cancer-related death worldwide [1,2]. Despite innovations in diagnostic testing, surgical technique, and development of new chemotherapeutic agents, five-year survival rate has not improved dramatically for patients with lung cancer [3,4]. Therefore, it is important to understand the molecular mechanisms involved in the pathogenesis and progression of metastasis to identify novel therapeutic targets and develop effective treatment strategies.

Deletion within the lung cancer tumor suppressor region at 3p21.3 constitutes the earliest premalignant chromosomal aberration in human lung cancers. Nineteen tumor suppressor genes (TSGs) reside to this region at 3p21.3. RBM5 maps to one end of this 19-gene deletion breakpoint, which is more frequently observed in tumors of lung, breast and kidney [28], and is not included in a slightly smaller 17-gene deletion that is common to breast and renal tumors [28]. There is increasing evidence suggesting that RBM5 plays an important role in lung cancer occurrence and development, nevertheless, there are few studies reporting on RBM5 expression in lung cancer tissues and tumor cell lines. In a very small cohort of eleven specimens, it was observed that the six tumor samples with the most significantly reduced RBM5 mRNA levels were of the squamous type [29-31] whereas three of the nine with the less significantly reduced RBM5 mRNA levels were adenocarcinomas. The one tumor sample with no change in RBM5 mRNA expression compared to its non-tumor tissue was an adenocarcinoma, while the one tumor sample that had more RBM5 mRNA than its non-tumor tissue was a large cell carcinoma [29,31]. In our current study, we found that the expression of RBM5 mRNA and protein were both significantly reduced in 120 cases of surgically resected NSCLC compared to the adjacent normal tissues, 30 of which were lung adenocarcinoma [32]. All the above suggest the critical and specific role of RBM5 in the development of lung cancer.

This report represents the first characterization of RBM5 tumor suppressive activity and its molecular mechanism in a lung adenocarcinoma animal model. We successfully established lung adenocarcinoma animal models *in vivo* on the back of nude mice. We found that RBM5 significantly inhibited the growth of lung adenocarcinoma *in vivo* by the induction of apoptosis. In Oh’s study, they also reported that RBM5 could inhibit the growth of human lung cancer A549 cells *in vitro* and *in vivo*[16]. The model they mentioned, however, was simply established using RBM5 stable transfected A549 cells to observe the growth retardation of tumor, without any metabolism for RBM5 in animals. *Salmonella enterica* ser. Typhi Ty21a has many of the desirable properties of a delivery vector, including targeting of multiple tumors from a distant inoculation site, selective replication within tumors, and the ability to express effector genes. *Salmonella enterica* ser. Typhi Ty21a has an excellent safety profile and a propensity for homing to tumors. In this study, we chose an efficient gene delivery system, attenuated *S. enterica* ser. Typhimurium, which could selectively target tumors and deliver the therapeutic agents into tumors while reducing the damage to normal tissue [23,33,34]. More recently, these bacteria have been engineered to express numerous genes, including survivin [35], MDM2 [36], Neu3 [37] and STAT3 siRNA [25] against prostate cancer in our laboratory. So this time, we first employed the attenuated *S. enterica* ser. Typhimurium to target lung tumors and directly deliver the therapeutic pcDNA3.1-RBM5 plasmids through the tail by intravenous injection.

Apoptosis is a complex, multistage process involving many genes. Among the wide spectrum of apoptosis regulatory proteins tested in our studies, the expression of proapoptotic proteins Bax, cleaved caspase-9, cleaved caspase-3, and cleaved PARP were found to be increased in RBM5-overexpressing tumor tissues, while the expression of apoptosis inhibitory protein Bcl-2 was observed at a lower level in RBM5-overexpressing tumor tissues. Thus, we conclude that RBM5 might induce apoptosis by triggering the mitochondrial apoptotic pathways, including caspase-9 and caspase-3 activations by means of increasing the expression of Bax and decreasing the expression of Bcl-2 [38]. In Oh’s study, mitochondrial apoptotic pathways initiated by Bax were also observed in the A549 cells transfected with RBM5 *in vitro*[16]. In addition, we also noted that increased expression of TNF-α and cleaved caspase-8 was associated with the RBM5 overexpression in tumor tissues. This finding, although it needs to be validated by further experiments, strongly suggested that RBM5 could initiate TNF-α-mediated apoptosis [39], in which capase-8, caspase-3 and PARP were activated. This potential apoptosis pathway induced by RBM5 could also be observed in human breast carcinoma MCF-7 cells [40,41] and leukemia Jurkat cells [42]. The current data suggest that although RBM5’s involvement in the death receptor-mediated apoptotic pathway is still to be investigated in depth, RBM5-mediated growth suppression, at least in part, employs regulation of the mitochondrial apoptotic pathways.

## Conclusions

In summary, we recently demonstrated that RBM5 overexpression can employ both TNF-α-mediated apoptotic pathways and mitochondrial apoptotic pathways to suppress the growth of lung adenocarcinoma, by establishing an implant tumor model *in vivo*, treated with attenuated *Salmonella*-RBM5, a plasmids delivery system. Our studies provide further data *in vivo* to explore the role and molecular mechanism of RBM5 in lung cancer pathogenesis and suggest a strategy for the development of rationally designed therapeutics using RBM5 as a target.

## Abbreviations

AC: Adenocarcinoma; CFU: Colony-forming unit; H&E: Hematoxylin and eosin; NSCLCs: Non-small cell lung carcinomas; PBS: Phosphate-buffered saline; SCC: Squamous cell carcinoma; SCLCs: Small cell lung carcinomas; RBM5: RNA-binding motif protein 5; SD: Standard deviation; siRNA: Small interfering RNA; TNF-α: Tumor necrosis factor alpha; TSG: Tumor suppressor gene; TUNEL: Terminal deoxynucleotidyl transferase (TdT)-mediated deoxyuridine 5'-triphosphate (dUTP) nick-end labeling.

## Competing interests

The authors declare that they have no competing interests.

## Authors’ contributions

CS performed all the experiments and drafted the manuscript. LZ participated in the analysis of TUNEL and H&E staining. SW and JZ contributed to the data analysis. BY and KW have contributed to the research design, and the data collection and interpretation. KW oversaw the design of the study and was involved in the critical revision of the manuscript. All authors have read and approved the final version of the manuscript.
